# A ‘good hospital’: Nurse and patient perceptions of good clinical care for HIV-positive people on antiretroviral treatment in rural Zimbabwe—A mixed-methods qualitative study

**DOI:** 10.1016/j.ijnurstu.2010.07.019

**Published:** 2011-02

**Authors:** Catherine Campbell, Kerry Scott, Claudius Madanhire, Constance Nyamukapa, Simon Gregson

**Affiliations:** a Health, Community and Development, Institute of Social Psychology, London School of Economics, London, United Kingdom; b Manicaland HIV/STD Prevention Project, Biomedical Research and Training Institute (BRTI), Harare, Zimbabwe; c Department of Infectious Disease Epidemiology, Imperial College, London, United Kingdom

**Keywords:** Antiretroviral treatment, HIV/AIDS, Nurse–patient interaction, Qualitative research, Resource-poor healthcare centres, Zimbabwe

## Abstract

**Background:**

Antiretroviral treatment for HIV is gradually being made available across sub-Saharan Africa. With antiretroviral treatment, HIV can be approached as a chronic, manageable condition rather than a shorter-term issue of palliative care. This treatment involves repeated interaction between health staff and patients for ongoing check-ups and prescription refills.

**Objective:**

This study aimed to understand patient and healthcare staff perceptions of good clinical antiretroviral treatment care.

**Design:**

Over 100 h of ethnographic observation at healthcare sites; interviews and focus groups with 25 healthcentre workers (mostly nurses), 53 HIV-positive adults taking ARVs and 40 carers of children on ART. The data were analyzed using thematic content analysis.

**Setting:**

Three healthcare sites providing free antiretroviral drugs in rural Zimbabwe, where the adult HIV infection rate is approximately 20%.

**Results:**

Contrary to reports of poor antiretroviral treatment adherence and task-oriented rather than patient-oriented nursing, our study found great patient commitment to adherence, outstanding nurse dedication and a pervasive sense of hope about coping with HIV. Within this context however there were some situations where patients and nurses had different expectations of the medical encounter, leading to stress and dissatisfaction. Patients and staff both emphasized the importance of nurse kindness, understanding, confidentiality and acceptance (i.e. treating HIV patients ‘like normal’) and patient adherence to medical directions. However, nurses at times overlooked the negative effects of long wait times and frequent hospital visits. Further, nurses sometimes conflated medical adherence with general patient obedience in all aspects of the nurse–patient relationships. Patients and staff were frustrated by the ambiguity and unpredictability surrounding key elements of hospital visits such as how much patients had to pay for service, how long it would take to be served, and whether drugs or the doctor's services would be available.

**What is already known about the topic?**

• Antiretroviral treatment (ART) is increasingly accessible to HIV-positive people in sub-Saharan Africa, primarily through resource-poor healthcare centres.
• ART requires long-term nurse–patient interaction for monitoring, adherence support and drug refill.

**What this paper adds**

• Contrary to concerns about non-adherence issues and high levels of nurse burn out, nurses and ART patients in rural Zimbabwe were positive about adherence levels and primarily pleased with the quality of care provided, despite staff and resource shortages.
• Nurse–patient interactions surrounding ART nonetheless present challenges to both parties that stem from differing struggles and priorities.
• These findings provide insights in the ongoing development of best practices in HIV nursing, with application to the general challenges of introducing new treatment protocols in resource-poor areas in relation to any health condition.

## 1 Introduction

AIDS is the biggest killer in sub-Saharan Africa (WHO, 2008a). In 2008 it accounted for 1.4 million deaths – 14.7% of total deaths – in the region (UNAIDS/WHO, 2009). HIV is highest among economically productive adults of childbearing age and has an enormous impact on families, communities, and regional economies. ART is gradually being made affordable and accessible to HIV infected people across the continent predominantly through the provision of free or highly subsidized antiretroviral (ARV) drugs in under-resourced medical settings (WHO, 2008b). ART is to be delivered through health centres as part of a package of care that includes co-trimoxazole prophylaxis, counselling, the management of opportunistic infections and comorbidities, and nutritional support. Following several counselling sessions, patients are initiated onto ART by a doctor. Nurses provide the majority of subsequent support through meeting with patients at the clinic on a regular basis.

ART roll-out presents new opportunities and challenges for healthcare providers, heralding a new era of HIV nursing in Africa. ART enables different types of relationships between nurses and patients, characterized by regular interactions over many years for check-ups and refills. Optimising the positive opportunities presented by ARVs requires a greater understanding of the changes they bring and the supports required by patients and nurses. We examine these issues through a case study of HIV-positive people receiving free ARVs through three healthcare centres in rural Zimbabwe. This paper explores how patients and nurses view the changes and challenges brought about by ART.

Zimbabwe was one of the first African countries to show a declining HIV rate (UNAIDS/WHO, 2005) with the adult prevalence rate falling from 25% in 1997 to 13.7% in 2009 (ZMoHCW, 2009). Despite major challenges in the early 2000s, ARVs have become increasingly accessible in Zimbabwe and by the end of 2009 the government succeeded in getting over 218,000 people on ART, 56% of those needing treatment (UNAIDS, 2010). Adherence throughout sub-Saharan Africa has been found to be high (Amberbir et al., 2008; Bisson et al., 2008; Orrell et al., 2003); a recent study of Ugandan and Zimbabwean ART patients found that good adherence increased from 87%, four weeks after ART initiation to 94% at 48 weeks, but only half the patients achieved good adherence at every visit in the first year (Muyingo et al., 2008).

Making ART accessible to the general population in sub-Saharan Africa is a new phenomenon presenting new challenges. With treatment, HIV can be considered a chronic disease requiring ongoing hospital visits and a carefully managed drug regime rather than a terminal illness requiring palliative care. A patient found HIV positive today, with access to ART, can expect to live 10 years or more (Walensky et al., 2009). With frequent health centre visits to renew prescriptions, ongoing interactions with healthcare workers are becoming a routine feature of many Africans’ lives.

ART has renewed nurses’ sense of hope but also increased their workloads (Stein et al., 2007). Overworked nurses who lack sufficient material and emotional support often fall back on task- rather than patient-oriented care (Fassin, 2008; Manongi et al., 2009). In the paper ‘Why do nurses abuse patients? Reflections from South African obstetric services’ Jewkes, Abrahams and Mvo report that the frequent and often violent abuse of patients can be caused by a complex interplay of concerns including organizational issues (such as a lack of nurse accountability), professional insecurities, a perceived need to assert control over the environment and sanctioning of the use of coercive and punitive measures to do so, and an underpinning ideology of patient inferiority compared to the nurses’ status as middle-class professionals (1998, p. 1781). Lewin and Green's 2009 paper ‘Ritual and the organization of care in primary care clinics in Cape Town, South Africa’ reports similarly fraught, although less overtly violent, patient–provider relationships. They found that healthcare providers used rituals (directly observed treatment of tuberculosis and daily prayer) to reinforce asymmetrical relations of power and to strengthen conventional modes of provider–patient interaction, characterized by rigid hierarchy. Such problematic relationships have presented significant barriers to the roll-out of new healthcare regimes in Africa, such as TB treatment (Lewin et al., 2005) and mental healthcare (Petersen, 1999), and could be a potential barrier to ART roll-out.

There has been little examination of the needs, expectations and interactions of ART patients and the nurses who care for them. Understanding what patients and nurses perceive to be good ART-related clinical care and exploring differences in these perceptions is a vital component of improving HIV care in poor countries. Most studies on patient–health worker interaction in the field of HIV focus on HIV/AIDS care before ARVs were widely available (e.g. Ehlers, 2006; Kohi and Horrocks, 1994). The studies that do examine clinical interactions surrounding ART are generally from rich countries (e.g. Wood et al., 2003) where ART has been accessible for significantly longer than in developing countries such as Zimbabwe. Research on ART in poor countries is very new and tends to focus primarily on issues of patient non-adherence (Rosen et al., 2007; Wringe et al., 2009) or explore the views of either health workers or HIV patients, rather than both (e.g. Wouters et al., 2008; Stein et al., 2007).

Improving the clinical experiences for patients and health staff in resource-poor environments is vital to support the ongoing response to HIV. Good quality patient–health worker relationships promote adherence (Deyo and Inui, 1980), with recent studies extending this finding to ART adherence (Roberts, 2002). Beyond adherence, the importance of positive clinical experiences, including positive interactions with health workers, is closely linked to patient willingness to pay for services (McPake, 1993) and nurse job satisfaction (Kangas et al., 1999).

It is less clear what factors lead to positive clinical experiences for patients and how more positive patient–health worker interactions can be fostered, particularly for patients on ART in resource-poor settings. Efforts to improve the nurse–patient interface frequently fail. For example, nurses in Tanzania recognized they were often rough with patients and provided slow service but suggested these problems stemed from complex issues surrounding low job satisfaction and resisted simplistic sensitivity training (Manongi et al., 2009). Manongi et al.’s study highlights the need for further research into the human dynamics of clinical service delivery and how clashing expectations and needs of staff and patients can be addressed. To this end, our paper will highlight how nurses and patients often have different goals in the medical encounter, leading to stress and misunderstanding.

## 2 Methods

Our qualitative research involved collaboration between two British universities and a Zimbabwean public health institute. Research was conducted by four experienced fieldworkers over six weeks in 2009 in rural Zimbabwe, focusing on three sites: a Catholic clinic, an Anglican hospital and a government hospital. Details of the region and health settings have been anonymised to protect the identity of participants.

### 2.1 Context

The HIV rate in the region is approximately 20% (Gregson et al., 2006). Residents of the region are primarily subsistence farmers. Most live in rural homesteads (compounds with several mud and thatch houses, a pit-latrine and animal pens), often without electricity. Large commercial farming estates in the region employ a significant portion of the local population. Many families have members working in major cities, some of whom send money back to the rural areas. Poverty is a major challenge and many local people receive food aid from international organizations.

All three health centres studied had infrequent electricity. The Catholic clinic relied on water from a nearby hand pump while the other two had running water, albeit irregularly. The government hospital had one staff doctor, almost 40 nurses and approximately 40 additional staff (nurse aids, counsellors, pharmacists, cleaners and administrators); the Anglican hospital had a clinical officer,^1^ about 50 nurses and about 30 additional staff. At both sites, the doctor/clinical officer also visited smaller clinics and attended meetings and workshops in the cities. The bulk of patient care was provided by nurses and counsellors. At the government hospital, the doctor had so many commitments outside the hospital that he was only able to treat hospital out-patients once every two weeks. The Catholic clinic was visited monthly by a doctor and was otherwise staffed by nurses.

### 2.2 Research design

The research involved interviews and focus groups with patients, carers of children on ART and healthcare providers (see Table 1), as well as ethnographic observation of treatment settings.

Interviews and focus groups were conducted with a total of 53 ARV users (19 one-hour interviews with people on ARVs, and four two-to-three-hour focus groups with eight to 10 participants) and 40 carers of children on ART (21 interviews and three focus groups). Since carers of children on ART must attend the clinic for their child's check-ups and prescription refills they have similar clinical experiences as adult ART patients. Most participants were recruited from openly HIV-positive community members known to the researchers through previous HIV/AIDS research. Others were approached as they visited hospital or clinic sites. A few participants approached the researchers asking to be interviewed because they had heard about the project. Researchers’ requests to interview a person on ART were only refused in one case, by someone who cited time limitations. Topic guides explored changing perceptions of HIV, social support and ways of coping with HIV and ART, issues surrounding treatment adherence, and experiences at the healthcare centre. During focus groups with patients, participants were invited to perform a role-play of ‘a good day at the clinic’ and ‘a bad day at the clinic’. These role-plays revealed a great deal about what makes clinical experiences positive or negative for ART patients.

Interviews and focus groups were conducted with a total of 25 health staff (nurses, HIV counsellors, pharmacists and a clerk) at the three hospital/clinic sites, and one focus group was held at the Anglican hospital. A focus group at the other two sites was not possible due to staff shortages. Staff members had often been born in the region or had moved there through marriage, sharing a common identity with patients. Researchers received permission to conduct the research from each health centre's doctor or nurse-in-charge. Staff members were then approached individually to participate; all agreed. To elicit information about challenges and positive elements of working with HIV/AIDS patients and issues of clinic access and treatment adherence, nurses were asked about their views of ‘good’ and ‘bad’ patients, sources of frustration and support in their lives and their perceptions of proper clinical care.

Over 100 h of ethnographic observation were conducted at the health centres, observing interactions as HIV patients waited for the doctor, paid hospital fees, visited the pharmacy, and waited for nurses to review their progress on ART and prescribe refills of their ARVs. Researchers did not observe private interactions between patients and staff. Observation focused on hospital activity, including interactions between patients and staff and the arrangement of people in hospital spaces. Extensive detailed notes were taken by the researchers throughout the hours of observation, recording what occurred (and when), how people were organized within the clinic space and comments made about the experience. These notes were included in the body of text data (along with focus group and interview transcripts) for analysis.

Data were collected by three Shona-speaking fieldworkers and a fourth researcher working with an interpreter. All audio files were translated into English and transcribed by trained researchers. To thank the informants, focus group participants were given soap, and interviewees were given a t-shirt.

### 2.3 Thematic analysis

The data were analyzed using thematic content analysis (Flick, 2006). Thematic network analysis is a process of encoding qualitative information to find, record and interpret patterns in ‘raw’ text (in this case transcripts of interviews and focus groups and ethnographic notes taken by researchers). Two researchers, working independently, carefully read and re-read the texts to find themes, called coding units, which describe and organize observations, or interpret aspects of the phenomenon (i.e. nurse–patient interactions and conceptions of ‘good clinical care’). The researchers then grouped codes into larger themes and compared their findings to ensure reliability. These larger themes were finally grouped into five central themes, which were: ideal interactions between nurses and patients, obedience vs. adherence, control of ARV pills, HIV clinic availability and ‘grey areas.’ These five themes provide the sub sections for the findings reported below.

## 3 Findings

Patients and nurses were overwhelmingly positive about ART and associated services. Many friendly staff–patients interactions were observed. Healthcare staff expressed great satisfaction at seeing patients improve and were extremely positive about adherence rates. They were also confident about the quality of their counselling services, often referring to its effectiveness in helping patients come to terms with the challenges of HIV and ART. Patients were happy to have access to free life saving drugs. Many spoke positively about the healthcare staff, the changes in their lives since beginning ARVs and their adherence.

Against this positive background, there were nonetheless challenges and difficulties, especially surrounding in-clinic experiences and interactions. Findings on patient and staff perceptions of good care were for the most part consistent between the health centres and are thus presented together; cases where significant differences arose are specified. One note on site differences concerns the role of religion in the clinic. Some patients reported finding the religious atmosphere enriching to their experience, as an ART patient explains:

There are times when the nurses come and greet everybody and invite us into a room. In that room we pray together…after that we will get served in a very friendly fashion (Patient, Anglican hospital, focus group (FG))

Staff at each site, however, generally favoured their own type of institution. Nurses at the Christian sites felt patients preferred religious-based care. Government hospital staff said secular care was better because it was free from church-related regulations.

It is better here because the church can provide spiritual counselling, which does not happen in government hospitals. (Nurse, Anglican clinic, interview)

I prefer working at a government hospital because the church hospital has too many church imposed regulations … here we only follow government regulations. (Nurse, government hospital, interview)

Whilst staff seemed to favour their own type of institution, many patients praised the encouragement and camaraderie fostered by church-based clinics.

### 3.1 Ideal interactions between nurses and patients

At all sites, patients and nurses spoke of the importance of kindness, understanding, confidentiality and stigma reduction. Basic friendliness came up repeatedly, with patients and nurses showing how a greeting from a nurse or an expression of gratitude from a patient was vital to positive clinical interactions. Beyond the frequently observed friendly greetings, patients and staff commented on the importance of these interactions:

We see the patients every month and serving them is a pleasure because they are always cheerful and they ask questions when they do not understand. They bring us bananas and whatever they have because we have built that relationship with them… just yesterday there is a guy who brought me some potato chips. (Pharmacist, government hospital, interview)

Both nurses and patients said listening was vital to good clinical care:

We listen to people's personal problems because HIV is more than a medical condition (Nurse, Anglican hospital, interview)

When they come here apart from getting their treatments they also want to be listened to, so we try to give them a chance to say what they want (Nurse, government hospital, interview)

I really appreciate their kindness and they always try to listen when we have issues. (Patient, Anglican hospital, interview)

Patients expressed dissatisfaction when nurses did not appear to listen to them:

Sometimes the nurse might just tell the patient to go on bed rest without even listening to what the patient has to say (Patient, Anglican hospital, FG)

If they ask what you are suffering from, and they think you are not a good patient, some nurses will not even listen to what I am trying to tell them. They will just go ahead and write what they think on my card (Patient, government hospital, FG)

Patients appreciated staff taking an interest in their lives and getting to know them over time. Nurses similarly emphasized that their main source of motivation was seeing patients improve over time and developing positive relationships with them. Nurses recognized the importance of being understanding about the difficult circumstances that many HIV patients were dealing with. One nurse explains her compassionate approach to patients who defaulted on appointments:

Suppose a patient has missed the review date. The reasons that they give for missing it makes you reverse your initial decision to feel frustrated or angry with them. One will tell you they couldn’t get transport, or they could not get the money to come, and maybe today they have borrowed money to be able to attend. For me to be dissatisfied with such a patient would mean that I was not doing justice to them. It's often their life situations that cause their behaviour. (Nurse, Anglican hospital, FG)

Almost all nurses said that maintaining confidentiality about patients’ HIV status was a core component of good nursing practice, expressing satisfaction with their and their colleagues’ ability to achieve this. In the face of high levels of HIV/AIDS stigma in the region, patients appreciated nurses treating them ‘normally’—like patients with different ailments. Handshakes featured prominently in accounts of positive nurse–patient relationships. Shaking hands is a culturally accepted greeting that enables nurses to show they are not afraid of touching HIV-positive patients.

### 3.2 Adherence versus obedience

Nurses and patients agreed that good clinical care included providing counselling and a supportive environment to help patients develop optimal adherence strategies. Adherence could be undermined by misunderstandings about drugs, side effects, and the practical challenges of taking ARVs correctly (including remembering to take medication and accessing food to eat with drugs). The importance of obeying counsellors’ and nurses’ directions around taking ARVs was emphasized by both patients and health staff. However, the line between adherence to medical guidance regarding taking ARTs, and general obedience to nurses was sometimes blurred. Patient deference to nurses on medical issues and the assertiveness of nurses on these same issues were appreciated by both parties. However, sometimes nurses appeared to require deference for its own sake, as a way of exerting authority or affirming their importance and superiority to patients, rather than for medical reasons.

Hospital staff were occasionally observed to make seemingly arbitrary demands that patients line up, stand or sit. Patients also commented on the difficulty of this type of interaction:

… He will be shouting different kind of instructions for example “make sure you are in line” and “may everyone sit down, I won’t serve anyone standing up”. The benches will be full so some will sit on the floor…if you try to complain he might even shout at you (Patient, government hospital, FG)

Power inequalities between patients and healthcare staff are often enacted and reinforced through the control of patient movement in hospital spaces (Jewkes et al., 1998). Telling a patient to sit down can be a kind act or, conversely, a means to display gratuitous control. Some patients referred to instances where they had felt disempowered by displays of power by health staff:

A ‘bad nurse’ may come at the bench and shout at those sitting there, saying: ‘You think I’m the one who caused you to get sick?’ and ‘You want to be helped?’ You just keep quiet but you won’t feel free (Patient, Catholic clinic, interview)

### 3.3 Conflicting perceptions surrounding how and when to access ART

The organization of ART programs served as the key area of misunderstanding between health workers and patients, and a key source of patient stress. When in good health, most patients sought to attend the clinic as seldom as possible for ARV refills and check-ups, and all wanted to receive reasonably prompt care during these visits. However the ART programs in this region usually require patients to come for monthly ARV refills, with all ART patients coming on specific days, leading to bottlenecks and slow care. The healthcare staff have reasons for this arrangement that are sometimes not understood or valued by patients; likewise it appears that the negative effects on patients of frequent, long visits are not always fully appreciated by staff. Conflicting expectations and desires surrounding monthly ARV drug refills and HIV clinic days are discussed in turn below.

#### 3.3.1 Monthly ARV refills

Patients and health staff had different perceptions of good care regarding how often patients should come to the hospital to report to nurses and get their next instalment of pills. Patients generally felt healthy and confident about their capacity to adhere. By contrast, nurses anticipated negative side effects and adherence problems, leading them to schedule frequent visits to monitor patient adherence and well-being. Most patients wanted several months’ supply of ARVs at a time, in a context where people often lived far from health centres and lacked money for transport. In addition, patients often battled to pay a one or two dollar^2^ consulting fee each time they visited the health centre. They also had to wait many hours in several queues to pay the consultation fee, be weighed and have their temperature taken, see the nurse and have the pharmacy fill their prescription. These health centre visits sapped their already limited economic and physical resources. Most patient informants were proud of their adherence rates, eager to emphasise their understanding of the importance of taking their drugs correctly:

I personally value my health, I love my family so much that I don’t want to die and leave them. So I religiously do what I am supposed to do to keep myself fit…My main motive is to follow all that I am advised to do so that I can look after my children (Patient, Anglican hospital, FG)

I am sure very few people are not taking drugs as directed, because these drugs will not just be given to anyone before they ascertain that you have been counselled and understand that this is a lifelong course. Nobody would wake up one day and say ‘I am feeling better now so I am stopping the drugs’. People really understand the value of taking these drugs as directed. (Patient, government hospital, FG)

Health staff had several reasons for prescribing one month of pills at a time. At times when ARVs were in short supply, staff had to give patients fewer pills and have them come back for refills more frequently. When supplies were adequate to give patients several months’ worth of pills, nurses usually still gave out only one month's supply in order to monitor adherence. To get their next instalment of pills, patients must show nurses the previous month's package of ARVs, which should have a few days’ worth of pill doses remaining. If the patient has more pills than he or she should, nurses know that the patient has skipped pills and can intervene to improve adherence. Nurses also use the monthly refill visits to ask patients about their health to track and deal with potential side effects or opportunistic infections. However, for stable patients with a record of high adherence who have been on ART for over six months, such regular check-ups can be relaxed, according to recent guidelines. The WHO's (2006) ART recommendations suggest that clinical monitoring for stable patients can be performed every six months.

Misunderstandings arise because some patients think a good nurse is one who prescribes several months of pills at a time and that a bad nurse or pharmacist is someone who gives out smaller amounts of pills:

One day we were discussing among ourselves after we were just given two months supply of ARVs, so we were very happy about it saying, these nurses have been good to us. (Patient, Catholic clinic, FG)

Patient 1: The nurses don’t agree to it [giving several months’ supply at once]. They only provide two months supply for people who will be travelling away for a long time, because they say they need to see us regularly

Patient 2: Even sometimes when the *nurses* prescribe a two months supply the *pharmacist* will just say “I will give you one month's supply”. (Patients, government hospital, FG)

In the following quotation, a patient suggests that she received fewer pills as punishment for lacking money to pay the consultation fee, despite the fact that the nurse explained there was a drug shortage:

The habit of nurses saying ‘there are no drugs’ happens if you don’t have the money [consultation fee]. Like the last time the doctor prescribed us a full month's supply of ARVs, but when we went to collect these from the nurse, she said ‘Is your doctor normal? How can he allow you to get so many tablets? Where does he think we get the drugs?’ And she gave a smaller supply, saying ‘these are the last pills we have in stock’. (Patient, Catholic clinic, FG)

As the following quotation illustrates, nurses and pharmacists frequently do not have the supplies for larger doses and feel that patients fail to understand this limitation.

Sometimes they just say they want a two months’ supply… they come up with all sorts of reasons and yet it affects us when we order from the AIDS and TB unit in Harare. They will not understand this. Some have got genuine reasons but some just want a two months’ supply just for the sake of it being possible (Pharmacist, government hospital, interview)

In this way, many staff felt that seeking to limit clinic visits was not, in itself, a credible reason for wanting several months dose at once—a patient must have some additional reason such as travelling out of the region.

Our study suggests that monthly refills deplete the energy of patients, involving patients in unnecessary and unaffordable expenditure on consultation fees and transport. However, in addition to drug supply irregularities, it seems that nurses perceive good clinical care to include notions of surveillance, in contrast to patients who see frequent visits as a hindrance to their well-being.

#### 3.3.2 HIV clinic availability

Another area of stress and conflict between patients and staff centred around long wait times at healthcare sites. Staff had several reasons for only offering ART review dates (check-up and refill times) on one or two days of the week, rather than spreading appointments throughout the week. Staff felt that focusing on particular health conditions on particular days was more efficient because it saves them from having to flip back and forth through different patient and drug registers. This arrangement also facilitates pharmacy efficiency, enabling them to package and account for the ARVs all at once. Nurses use the quieter, non-ART days to catch up on paperwork.

In contrast, patients dread these ART days. Since they are seen on a first-come-first-served basis people must arrive extremely early in the morning and wait for hours in very long queues, often of up to 40 people. A sense of fear and dread pervades these queues as patients scramble to keep their place in line and worry about not seeing the nurse before closing hours. Patients occasionally tried to avoid the long queues by coming on non-ART days:

Some people do avoid coming on their review dates because they know the queue will be very long here so they would come two or three days later when the clinic will not be very busy (Nurse, government hospital, interview)

Patients reported that if they try to avoid the long queue by coming on a non-ART day the nurses demand an appropriate explanation (such as illness or funeral attendance), and that they seldom ‘got away with this’ more than once.

While some nurses mentioned the importance of seeing patients in a timely manner, most appeared not to appreciate the severe discomfort associated with these queues and waiting times for each refill. Staff occasionally even suggested that dedicated ART days benefited patients, enabling them to meet with one another and discuss their condition:

When they come, they sit on the benches and start to discuss their issues. Sometimes we would be calling out names yet they would be busy discussing their issues. They are so happy one of them said when we meet we have a lot to share and this disease has created friends for us… it is good that they have their special day, they meet and discuss all their issues… I am sure they are keen to come here and spend the whole day discussing their issues (Nurse, government hospital, interview)

This sentiment failed to resonate with ART patients who felt fast service was central to good care and who have plenty of opportunities to discuss their conditions in local HIV support groups.

### 3.4 Grey areas surrounding payments and access to service

‘Grey areas’ in policy (i.e. where rules exist but are widely known to bend) lead to stress, take up time and foster negative interactions between staff and patients. There are two clear examples of these problematic grey areas, where patients and staff have different expectations and needs which are mediated by ambiguous rules: payment for medicine and consultation, and access to medical service.

As mentioned above, each site charges a one or two dollar consultation fee. In addition, all medication other than ARVs and co-trimoxazole (a pre-ARV), such as drugs for opportunistic infections, must be purchased by patients, since the hospitals do not receive these medicines free through government or charity organizations. However, having to pay is not an unbendable rule. Sometimes patients are denied service or drugs if they cannot pay; other times, the fee is waived or turned into a debt. Sometimes patients are referred to the accounts department and after negotiation are given permission to receive service or drugs for free. Patients often lamented that, regardless of whether fees were completely waived or not, they were subject to harsh, off-putting treatment if they could not pay up-front:

They will say ‘How can you just walk up here and say stamp my card without money?’ …They say ‘You have to pay the dollar’. And when we say ‘No but we don’t have the money’, they respond ‘so do you want me to pay for you?’ Then they refer us to the accounts section. So even if we go on to get good service from the nurse we would have been destabilized already (Patient, government hospital, FG)

Patients and staff both recognized a lack of clear guidelines on the procedure for patients who cannot pay; it often depends on the individual staff members working that day or the persuasiveness of the patients. This ambiguity leads to many stressful interactions where patients plead for free care or medicine and nurses try to balance their desire to help to poor patients with the need to collect revenue for the health centre:

We are also human beings so we hear all these stories, people come with all their different stories and excuses on why they should be exempted from paying for their tablets. Some of these stories also cause us a great deal of stress (Nurse, Anglican hospital, interview)

In a role-play performed by ART patients in a focus group, participants portrayed a nurse trying to send away someone who could not afford the consultation fee. But after further pleading, she relented. Another patient describes how a ‘good nurse’ will coach the patient on how best to present his or her case to the administration, for example by advising them to ask for the fee to be made into a debt rather than waived.

The ambiguity surrounding payment for drugs and consultation causes stress not only between patients and staff but also within different departments of the hospital. When nurses or clerks, who control up-front payments, waive fees they often face reprimands from the accounts department. In the following quotation a nurse explains this tension:

With accounts I think understanding each other is difficult. We need money when we are at accounts. So if a patient comes and you give her credit, then she comes again you give her another credit, the administrator will disapprove of that (Nurse pharmacist, Anglican hospital, FG)

Access to service presents another area that leads to conflict. There is a general understanding that the doctor and nurses are seen on a first-come-first-served basis. However, this system is occasionally circumvented, particularly at the government hospital and particularly when waiting for the doctor, where the stakes are higher than waiting for nurses since the doctor is available so rarely. Doctor hours are limited and there are often lines of over 40 people to see the doctor in a 2-h window. Patients are sometimes advanced in line by nurses because they are related to the nurse, due to severity of the illness, or because they are with children, as the quotation below discusses.

As you might know people have become very corrupt. If you have relatives working there, then you will avoid all these queues and just come in front and get served while we spent the whole day waiting. (Patient, government hospital, FG)

Since the justifications for changing the order are flexible, patients feel very worried about losing their position and sometimes try to assert their position in line aggressively.

## 4 Conclusion

This paper has explored the perspectives of ART patients and nurses on what constitutes good clinical care in the context of ongoing HIV/AIDS management through ARV provision. We have sought to illustrate how conflicting expectations in ART-related clinical encounters can lead to stressful and unsatisfying nurse–patient interactions. To this end, we have presented findings that detail specific areas where nurses and patients hold different conceptions of good clinical care and different priorities for clinical interactions.

Healthcare staff often sought evidence of patient obedience and respect. Whilst nurses seemed to regard these displays as facilitators of adherence and service efficiency, patients often found these demands for obedience disheartening. In some cases, nurses also appeared to have sought evidence of their power over patients in order to cope with the day-to-day stress and disempowerment of working in resource limited and somewhat unpredictable environments. As mentioned in the introduction, attempts to use sensitization training to address abusive and disrespectful staff behaviour in resource-poor settings have proved challenging (e.g. Manongi et al., 2009). Without addressing root issues, such as chronic stress surrounding shortages of staff, drugs and equipment alongside a lack of respect between different healthcare cadres and low staff accountability, it appears difficult to change healthcare worker behaviour towards patients. However, the fact that high quality care dominated in our study and that many of the instances of problematic staff–patient interaction were linked more to differing expectations than ill-will shows that resource limitations and compassionate nursing can go hand-in-hand. Greater insight into the factors that facilitate such compassionate nursing will help support future efforts to foster positive staff–patient relationships in resource-poor settings.

Patients favoured an ART program where visits to the hospital or clinic were quick and less frequent. Nurses frequently overlooked or failed to concern themselves with the importance of fast service and the difficulties patients faced to attend hospital frequently; instead, they prioritized careful, systematic and regular adherence surveillance and evidence of patient respect. Finding strategies to speed up patient visits on review days (perhaps by reviewing ART on more than one or two days a week or increasing staff on high-capacity days) would go a long way towards alleviating a key source of patient stress. Ambiguity surrounding various elements of hospital administration (especially paying for services/drugs and order of access to the doctor and nurses) lead to prolonged negotiations between staff and patients. Considering that almost every patient is poor and in serious need of nurse or doctor assistance, staff members struggle to choose who to assist financially and who to prioritize in line. Add to that the pressure to help relatives and friends and it becomes clear that healthcare staff deal with many conflicting demands on a regular basis at work, for example, between meeting patient needs and generating much needed revenue for their health centre.

Here we re-emphasise that the challenges facing staff and patients as ART becomes a lifelong reality for tens of thousands of Zimbabweans must be understood in the context of the overwhelmingly positive reception of ART in the region. Overall, patients were extremely optimistic about their prospects of living on ART and their capacities to adhere to treatment. Praise for staff kindness were far more commonplace than any type of complaint, a great testament to the commitment and capacities of these health staff who work in severely under-resourced settings. Likewise, staff were overwhelmingly optimistic about ARVs and expressed confidence in their ability to provide good quality care.

This case study takes into account the views of both patients and healthcare providers to explore the challenges of ART provision in a resource-poor setting. We have sought to develop a more complete picture of why nurses and patients behave the ways they do and how their differing priorities play into their levels of satisfaction with clinical interactions. Our findings contribute to the literature on best practice in the era of ART by suggesting that, contrary to the more common research concerns about non-adherence and nurse burn out, clinical care that satisfies ART patients and nurses hinges on finding ways to understand and address differing nurse and patient needs and priorities.

The role of underlying Christian values and links to churches warrants further investigation as findings from this study suggest that patients appreciate the spiritual dimension of the care and sense of solidarity offered by Christian healthcare facilities. The fact that ART was relatively new to our study sites (having begun slightly over a year prior to the study) limits our understanding of the longer-term evolution of patient-provider relationships in the era of ART. A follow-up study in 2012 that revisits the same healthcare sites plans to provide further insights into the long-term clinical interactions necessitated by ART. Such a study will seek to further our understanding of HIV nursing in the era of ART by illuminating whether our findings reflect the ongoing practice of ART clinical care or initial excitement and uncertainty related to the early stages of ART roll-out.

In the context of our interest in the implications of ART roll-out in sub-Saharan Africa and the special challenges posed by delivering this treatment in resource-strapped settings, this paper has reported on a multi-method qualitative study of ART provision in rural Zimbabwe. ART roll-out across sub-Saharan Africa presents unique opportunities and challenges for the nursing profession, enabling HIV care to evolve from a primarily opportunistic infection and palliative approach to a long-term chronic illness management approach. Our findings add to the global discussion of best practice in HIV care by highlighting the ways in which contrasting priorities of patients and nurses can lead to dissatisfaction.

## Funding source

This work was supported by the Wellcome Trust.

## Role of the funding source

The Wellcome Trust played no role in the conduct of the research or the preparation of the article.

## Conflict of interest

None declared.

## Ethical approval

Ethical approval for the study was granted by the Medical Research Council of Zimbabwe (Ref: A/681) and the Imperial College Research Ethics Committee (Ref: ICREC_9_3_13).

## Figures and Tables

**Table 1:** Summary of study participants.

	Participants	Interviews	FGD
Staff	25	18	1
Carers of children	40	21	3
Patients	53	19	4
Total	118	58	8

## Footnotes

1 A clinical officer is healthcare worker who is trained for three years and received a diploma in community health, surgery and community medicine. Clinical officers, unlike nurses, are licensed to prescribe medicine and initiate ART in Zimbabwe.

2 In 2008, the Zimbabwe government switched to a multi-currency system within which the US dollar is the most widely used currency.

## References

[bib0005] Amberbir A., Woldemichael K., Getachew S., Girma B., Deribe K. (2008). Predictors of adherence to antiretroviral therapy among HIV-infected persons: a prospective study in Southwest Ethiopia. BioMed Central Public Health.

[bib0010] Bisson G.P., Rowh A., Weinstein R., Gaolathe T., Frank I., Gross R. (2008). Antiretroviral failure despite high levels of adherence: discordant adherence–response relationship in Botswana. Journal of Acquired Immune Deficiency Syndrome.

[bib0015] Deyo R.A., Inui T.S. (1980). Dropouts and broken appointments. A literature review and agenda for future research. Med Care.

[bib0020] Ehlers V.J. (2006). Challenges nurses face in coping with the HIV/AIDS pandemic in Africa. International Journal of Nursing Studies.

[bib0025] Fassin D. (2008). The elementary forms of care: an empirical approach to ethics in a South African hospital. Social Science & Medicine.

[bib0030] Flick U. (2006). An Introduction to Qualitative Research.

[bib0035] Gregson S., Garnett G., Nyamukapa C., Hallett T., Lewis J., Mason P., Chindiwana S., Anderson R. (2006). HIV decline associated with behaviour change in rural Zimbabwe. Science.

[bib0040] Jewkes R., Abrahams N., Mvo Z. (1998). Why do nurses abuse patients? Reflections from South African obstetric services. Social Science and Medicine.

[bib0045] Kangas S., Kee C., McKee-Waddle R. (1999). Organizational factors, nurses’ job satisfaction, and patient satisfaction with nursing care. Journal of Nursing Administration.

[bib0050] Kohi T.W., Horrocks M.J. (1994). The knowledge, attitudes and perceived support of Tanzanian nurses when caring for patients with AIDS. International Journal of Nursing Studies.

[bib0055] Lewin S., Dick J., Zwarenstein M., Lombard C. (2005). Staff training and ambulatory tuberculosis treatment outcomes: a cluster randomized controlled trial in South Africa. WHO Bulletin.

[bib0060] Lewin S., Green J. (2009). Ritual and the organization of care in primary care clinics in Cape Town, South Africa. Social Science & Medicine.

[bib0065] Manongi R., Nasuwa F., Mwangi R., Reyburn H., Poulsen A., Chandler C. (2009). Conflicting priorities: evaluation of an intervention to improve nurse–parent relationships on a Tanzanian paediatric ward. Human Resources for Health.

[bib0075] McPake B. (1993). User charges for health services in developing countries: a review of the economic literature. Social Science and Medicine.

[bib0080] Muyingo S.K. (2008). Patterns of individual and population-level adherence to antiretroviral therapy and risk factors for poor adherence in the first year of the DART trial in Uganda and Zimbabwe. Journal of Acquired Immune Deficiency Syndromes.

[bib0085] Orrell C., Bangsberg D.R., Badri M. (2003). Adherence is not a barrier to successful antiretroviral therapy in South Africa. AIDS.

[bib0090] Petersen I. (1999). Training for transformation: reorientating primary health care nurses for the provision of mental health care in South Africa. Journal of Advanced Nursing.

[bib0095] Roberts K.J. (2002). Physician–patient relationships, patient satisfaction, and antiretroviral medication adherence among HIV-infected adults attending a public health clinic. AIDS Patient Care and STDs.

[bib0100] Rosen S., Fox M., Gill C. (2007). Patient retention in antiretroviral therapy programmes in sub-Saharan Africa: a systematic review. PLoS Medicine.

[bib0105] Stein J., Lewin S., Fairall L. (2007). Hope is the pillar of the universe: health-care providers’ experiences of delivering ART in primary health-care clinics in the Free State province of South Africa. Social Science and Medicine.

[bib0110] UNAIDS/WHO (2005). Evidence for HIV Decline in Zimbabwe: A Comprehensive Review of the Epidemiological Data.

[bib0120] UNAIDS/WHO, 2009. AIDS epidemic update. http://data.unaids.org/pub/Report/2009/JC1700_Epi_Update_2009_en.pdf.

[bib0165] UNAIDS. Zimbabwe - 2010 Country Progress Report Geneva: UNAIDS http://data.unaids.org/pub/Report/2010/zimbabwe_2010_country_progress_report_en.pdf (retrieved on 14 August 2010); 2010.

[bib0125] Walensky R., Wolf L., Wood R., Fofana M., Freedberg K., Martinson N., Paltiel D., Anglaret X., Weinstein M., Losina E. (2009). When to start ART in resource-limited settings. Annals of Internal Medicine.

[bib0130] WHO, 2006. ART for HIV infection in adults and adolescents: recommendations for a public health approach. 2006 revision. Strengthening health services to fight HIV/AIDS. http://www.who.int/hiv/pub/guidelines/artadultguidelines.pdf.

[bib0135] WHO (2008). The Global Burden of Disease: 2004 Update. http://www.who.int/healthinfo/global_burden_disease/GBD_report_2004update_full.pdf.

[bib0140] WHO (2008). Towards Universal Access: Progress Report 2008. http://www.who.int/hiv/pub/towards_universal_access_report_2008.pdf.

[bib0145] Wood E., Montanera J., Bangsbergc D., Tyndalla M., Strathdeed S., O'Shaughnessya M., Hogga R. (2003). Expanding access to HIV ART among marginalized populations in the developed world. AIDS.

[bib0150] Wouters E., Heunis C., Van Rensburg D., Meulemans H. (2008). Patient satisfaction with antiretroviral services at primary health-care facilities in the Free State, South Africa. BMC Health Services Research.

[bib0155] Wringe A., Roura M., Urassa M., Busza J., Athanas V., Zaba B. (2009). Doubts, denial and divine intervention: understanding delayed attendance and poor retention rates at a HIV treatment programme in rural Tanzania. AIDS Care.

[bib0160] ZMoHCW (Zimbabwe Ministry of Health and Child Welfare) (2009). Zimbabwe National HIV and AIDS Estimates.

